# The Registration of Midwives

**Published:** 1891-01-03

**Authors:** 


### 1890: A Various Year.

Never perhaps in the history of the world did the affairs of sick persons, of nurses, doctors, and hospitals engage so much of the attention of a busy world as during the year 1890. In January, London and other English towns began to realise that Influenza was something more than a name. The men and women of this generation hardly know what a serious epidemic is. Smallpox in an epidemic form is a thing of the past; and many are beginning to believe that cholera never need be seriously dreaded again. But the influenza epidemic, which injured so many and killed so few, gave to the London world a small taste of what "plague, pestilence, and famine" may mean to a crowded population. Serious apprehensions were entertained that a recrudescence of the disease was about to be witnessed during the present winter, but, thanks apparently to the chill frosts and nipping blasts of an extraordinarily cold December, those apprehensions have not been realised. Perhaps we are thence entitled to conclude that, whatever may have been the origin of last winter's influenza, the unusual quantity of rain which fell, and the persistently damp cold of the atmosphere, had much to do with it.

### THE REGISTRATION OF MIDWIVES.

The month of January witnessed an important movement on behalf of poor lying-in women. Those women pass through the period of nature's agony with little help, and that often of the most ignorant kind. This should not be: it is a scandal to an intelligent and Christian country. Steps were taken in the early part of the year to get a measure passed through Parliament for the registration of midwives. The object of that measure was to insure at least a moderate amount of trained intelligence and skill on the part of those who are permitted to attend poor women in confinement. Such a measure cannot possibly injure anybody, except by taking a few small fees out of the pockets of medical practitioners. But let it be said to the honour of the medical profession that it has loyally supported the Midwives Registration Bill. Parliament, being informed of this truly worthy and philanthropic attitude on the part of the medical profession, ought not to ask a single further question on the subject, but to transfer the measure to the statute book without delay.

